# Right ventricle dysfunction assessment for transcatheter tricuspid valve repair: A matter of debate

**DOI:** 10.1111/eci.13653

**Published:** 2021-07-26

**Authors:** Alberto Preda, Francesco Melillo, Luca Liberale, Fabrizio Montecucco, Eustachio Agricola

**Affiliations:** ^1^ Cardiovascular Imaging Unit IRCCS San Raffaele Scientific Institute Milan Italy; ^2^ Department of Internal Medicine First Clinic of Internal Medicine University of Genoa Genoa Italy; ^3^ Center for Molecular Cardiology University of Zürich Schlieren Switzerland; ^4^ IRCCS Ospedale Policlinico San Martino Genoa–Italian Cardiovascular Network Genoa Italy; ^5^ Vita‐Salute San Raffaele University Milan Italy

**Keywords:** echocardiography, right ventricle dysfunction, right ventricle function assessment, strain imaging, TAPSE, transcatheter tricuspid valve repair

## Abstract

Newer approaches in transcatheter tricuspid valve replacement (TTVR) have recently showed optimistic data of efficacy and safety in patients at high risk for surgery. However, the absence of residual regurgitation (and subsequently higher likelihood for developing afterload mismatch) with TTVR compared with transcatheter tricuspid valve intervention may become a critical concern if RV dysfunction is misdiagnosed. Indeed, such sudden increase in afterload on the right ventricle (RV) may not be tolerable, resulting in higher risk of acute right heart failure in the early postoperative period. In this context, strain imaging may find a further application to provide a more comprehensive stratification of the severity of RV dysfunction and thus help to better define the eligibility criteria and timing for TTVR. Meanwhile, it is of paramount importance to underline the contribution given by the Trivalve study on the understanding of the role of RV function in TTVI, that so far was largely undefined, being evaluated only in small noncontrolled cohorts.

Significant tricuspid regurgitation (TR) is currently the fourth most frequent isolated valvular disease and is often found in association with other valvular defects.^1^ TR increases cardiovascular mortality and recurrent hospitalizations for heart failure and reduces quality of life.^2^, ^3^ Accordingly, TR is often repaired at the time of concomitant surgery for left valvular diseases.^4^, ^5^ Indeed, most cases of significant TR are secondary and are due to annular dilation and/or leaflet tethering in the setting of RV remodelling due to pressure or volume overload.^6^ This mechanism results from a complex and high dynamic interplay among different structures including valve apparatus, right ventricle (RV), lungs and left heart.^6^, ^7^ Mortality for isolated tricuspid valve surgery is reported higher than that of any other single heart valve surgery^8^ and in those patients with contraindications for surgery, transcatheter tricuspid valve intervention (TTVI) has been performed so far on a compassionate use.^9^, ^10^ However, TTVI is still developing and evidence from nonrandomized controlled trial revealed an association of TTVI with improved survival in patients with symptomatic severe TR in comparison with conservative management.^11^ According to the most recent guidelines,^12^, ^13^ RV dysfunction is an important negative prognostic factor for surgical intervention and impacts on patient eligibility to surgery. Indeed, RV dilatation and dysfunction are part of the vicious circle that maintain and promote TR overtime and might cause repair failure. However, the prognostic role of RV dysfunction beyond the beneficial effect of repair is still a matter of debate. Therefore, the attention in the recent years has been focused on identifying the correct timing of intervention, with the aim to grant an effective and stable correction of valvular defect, potentially reversing right cardiac remodelling and improving outcomes eventually.

Recently, the results of the largest study investigating the 1 year outcome of TTVI in relation to different degrees of RV function were published in *Eurointervention*.^14^ Patients undergoing TTVI were enrolled from the Trivalve Registry, including data from 21 heart centres in Europe and North America. All patients (n = 426) had severe or greater symptomatic TR (NYHA III/IV) and underwent TTVI on an off‐label or compassionate basis according to local multidisciplinary team decision. A residual TR ≤2 was set among the criteria to define a procedural success. Patients were matched 1:1 with conservatively managed patients using propensity scores based on different variables including tricuspid annular plane systolic excursion (TAPSE), age, EuroSCORE II, estimated glomerular filtration rate, left ventricular ejection fraction and end‐diastolic diameter, NYHA class, presence of atrial fibrillation/flutter and systolic pulmonary artery pressure. Patients were then stratified into three groups according to the degree of RV function measured with TAPSE (TAPSE >17 mm: preserved, TAPSE 13‐17 mm: mid‐range and TAPSE <13 mm: reduced). At the end of the 1 year follow‐up, all‐cause mortality (primary end point) reached 25.4% in controls vs 13.1% in TTVI cohort (*P* = .031), with a clear benefit in terms of reduced mortality in case of successful intervention (*P* = .007). With respect to the relationship between RV function and TTVI, the subgroup with mid‐range reduction in RV function showed a better survival compared to groups with preserved and reduced RV function (*P* = .004). Upon multivariate analysis, only procedural success (HR 0.22; 95% CI: 0.09, 0.57) and EuroSCORE II (HR 1.10; 95% CI: 1.01, 1.20) remained independent predictors of an adverse outcome in TTVI population. Some limitations are reported from the authors: the lack of randomization was partially corrected by matching with the use of a propensity score, making the population more homogeneous than previous similar studies. Nevertheless, such an approach does not fully protect from unidentified confounders. The cohort also included patients with significant mitral regurgitation treated with transcatheter mitral valve intervention at the same time of the tricuspid repair while the same patients in the control group were excluded. The control group therefore missed an entire population of patients at higher risk of RV dysfunction and post‐repair TR recurrence. Similarly, also patients with congenital TR were not included in the cohort. As a further limitation, rate of rehospitalization for heart failure or improvement in functional class was not evaluated. Such data would have been useful both to better understand the reasons underlying the improvement of prognosis of the group with mid‐range RV dysfunction and to better delineate the effect of TTVI in patients other than mid‐range RV dysfunction. Figure 1

These results may contrast with what reported by Miura et al^15^ who identified right ventricular dysfunction as an independent predictor of all‐cause mortality and heart failure rehospitalization in patients treated with TTVI. However, the nonlinear relationship of TTVI outcome and TAPSE is an interesting finding and highlights an important unresolved question. Indeed, the partition in the three groups of the study population is affected by the strong limitation of using a single, surrogate parameter of the RV function‐like TAPSE. Because TAPSE is an established echocardiographic parameter used to measure RV function, with discrete accuracy and reproducibility compared with CMR,^16^ it has several not negligible limitations: (1) the assumption that free wall motion reflects global function; (2) its absolute value is highly dependent on the angle of the M‐mode cursor over the tricuspid annulus; (3) TR with preserved systolic function TAPSE might have supra‐normal values (due to a hyperdynamic volume loaded RV); thus, measurements falling into the normal range indicate RV dysfunction; and (4) it still lacks data on its prognostic value during earliest stages of RV dysfunction. Under this point of view, the quest is open for widely accessible methods to assess RV function in the setting of TR with high sensitivity to identify early dysfunction. Recently, the application of deformation imaging to assess RV free wall longitudinal strain has been shown to identify higher rates of RV dysfunction in patients with significant TR and to be associated with worse outcome beyond conventional echocardiography in this population.^17^ According to these results, Ancona et al recently confirmed the predictive (in terms of detection of early RV dysfunction) and prognostic value of RV strain analysis in a population with severe degrees of TR.^18^ Moreover, in this study 42% of patients with reported normal RV function at conventional echocardiography was reclassified as RV dysfunction according to strain analysis. It would be interesting to know the effect of strain analysis on patient classification in the cohort of the Trivalve Registry especially in those showing normal RV function or mid‐range RV dysfunction. Although cardiac magnetic resonance (CMR) is considered the gold standard for RV quantification, CMR‐specific cut‐off values for TR severity are not yet defined.^19^ So far, only one study^20^ investigated the prognostic role of volumetric RV quantification via CMR on the risk of post‐TR surgery mortality while none investigated the prognostic role of TR quantification with such an imaging technique.

Finally, newer approaches in transcatheter tricuspid valve replacement (TTVR) have recently showed optimistic data of efficacy and safety in patients at high risk for surgery.^21^ However, the absence of residual regurgitation (and subsequently higher likelihood for developing afterload mismatch) with TTVR compared with TTVI may become a critical concern if RV dysfunction is misdiagnosed. Indeed, such sudden increase in afterload on RV may not be tolerable, resulting in higher risk of acute right heart failure in the early postoperative period. In this context, strain imaging may find a further application to provide a more comprehensive stratification of the severity of RV dysfunction and thus help to better define the eligibility criteria and timing for TTVR. Meanwhile, it is of paramount importance to underline the contribution given by the Trivalve study on the understanding of the role of RV function in TTVI, that so far was largely undefined, being evaluated only in small noncontrolled cohorts^22^

## CONFLICT OF INTEREST

None.

1

**FIGURE 1:**
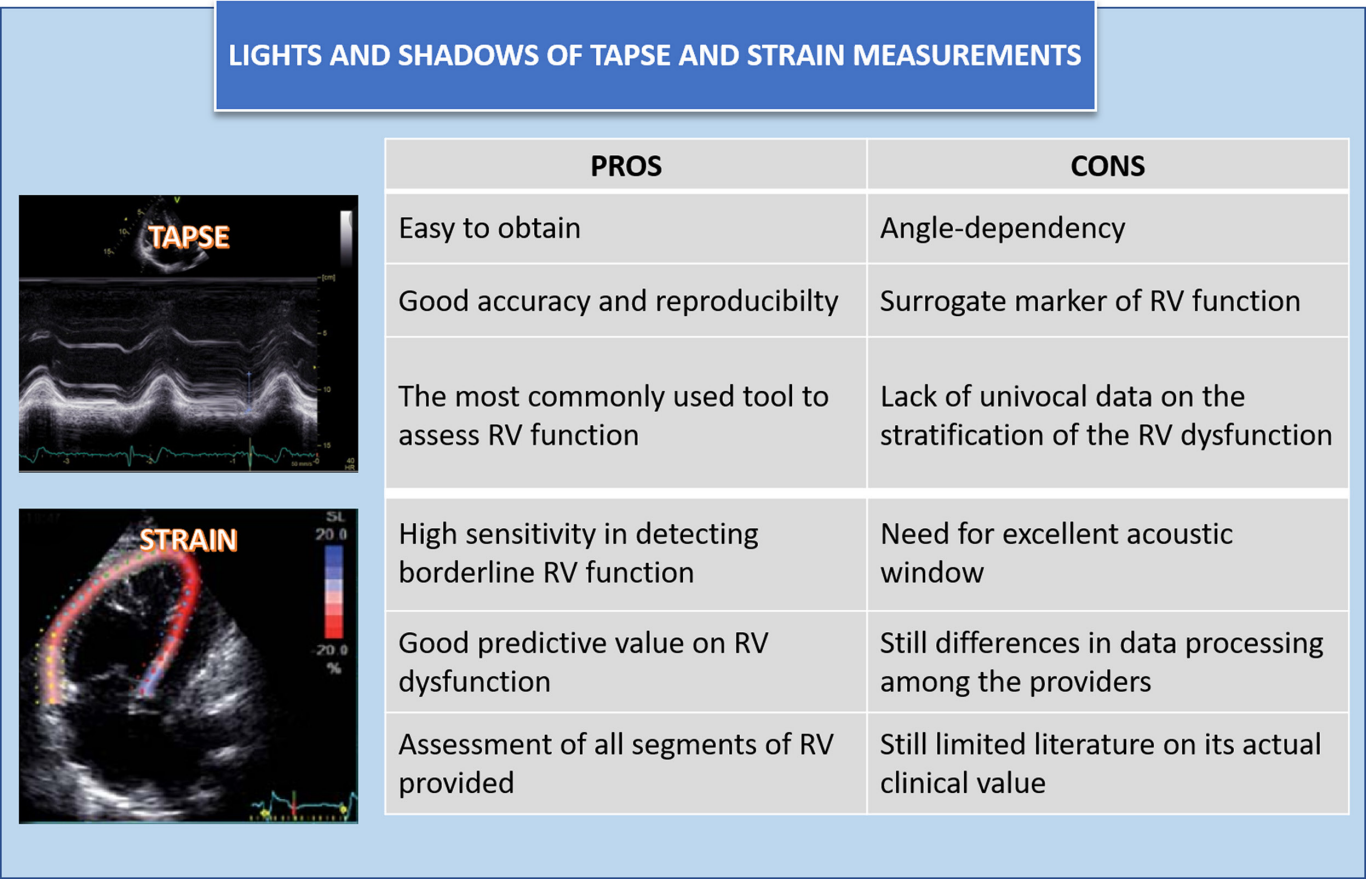
Lights and shadows of TAPSE and STRAIN measurements. Old and new echocardiographic methods for the assessment of the RV contractile function are compared, illustrating their strengths and weaknesses
